# Effectiveness of Olympic Combat Sports on Balance, Fall Risk or Falls in Older Adults: A Systematic Review

**DOI:** 10.3390/biology11010074

**Published:** 2022-01-04

**Authors:** Pablo Valdés-Badilla, Rodrigo Ramirez-Campillo, Tomás Herrera-Valenzuela, Braulio Henrique Magnani Branco, Eduardo Guzmán-Muñoz, Guillermo Mendez-Rebolledo, Yeny Concha-Cisternas, Jordan Hernandez-Martínez

**Affiliations:** 1 Department of Physical Activity Sciences, Faculty of Education Sciences, Universidad Católica del Maule, Talca 3530000, Chile; 2 Escuela de Educación, Universidad Viña del Mar, Viña del Mar 2520000, Chile; 3 Department of Physical Activity Sciences, Universidad de Los Lagos, Santiago 5290000, Chile; r.ramirez@ulagos.cl; 4 Exercise and Rehabilitation Sciences Laboratory, School of Physical Therapy, Faculty of Rehabilitation Sciences, Universidad Andres Bello, Santiago 8370035, Chile; 5 Department of Physical Activity, Sports and Health Sciences, Faculty of Medical Sciences, Universidad de Santiago de Chile (USACH), Santiago 8370003, Chile; tomas.herrera@usach.cl; 6 Graduate Program in Health Promotion, Cesumar University (UniCesumar), Maringá 87050-900, Brazil; braulio.branco@unicesumar.edu.br; 7 Escuela de Kinesiología, Facultad de Salud, Universidad Santo Tomás, Talca 110231, Chile; eguzmanm@santotomas.cl (E.G.-M.); gmendez001@gmail.com (G.M.-R.); yenyf.concha@gmail.com (Y.C.-C.); 8 Pedagogía en Educación Física, Facultad de Educación, Universidad Autónoma de Chile, Talca 3467987, Chile; 9 Universidad de los Lagos, Osorno 5290000, Chile; jordan.hernandez@ulagos.cl

**Keywords:** martial arts, combat sports, postural balance, accidental fall, aged, musculoskeletal and neural physiological phenomena

## Abstract

**Simple Summary:**

The regular practice of physical activity has achieved greater diffusion in recent years as a strategy for fall prevention and improving or maintaining the general health status of older adults, which contributes to an active and healthy lifestyle. The strategies to promote active and healthy aging need to be reassessed due to the COVID-19 pandemic to promote the safe practice of physical activity among older adults, considering, among other measures, practicing social distancing or training at home. Such a scenario could provide the opportunity to promote physical activity that requires a limited number of participants in reduced spaces for its practice, such as Olympic combat sports. In this review, an overview of the effects of Olympic combat sports on balance, fall risk, or falls in older adults is presented. From 1496 records, eight studies were included, involving 322 older adults (64% female; mean age = 71.1 years). The available evidence does not allow a definitive recommendation for or against Olympic combat sports interventions as an effective strategy to improve balance and reduce the fall risk or falls in older adults. Therefore, more high-quality studies are required to draw definitive conclusions.

**Abstract:**

This systematic review and meta-analysis aimed to assess the available body of published peer-reviewed articles related to the effects of Olympic combat sports (OCS), compared with active/passive controls, on balance, fall risk, or falls in older adults. The TESTEX and GRADE scales assessed the methodological quality and certainty of the evidence. The protocol was registered in PROSPERO (code: CRD42020204034). From 1496 records, eight studies were included, involving 322 older adults (64% female; mean age = 71.1 years). The TESTEX scale revealed all studies with a score ≥ 60% (moderate-high quality). The GRADE scale indicated all studies with at least some concerns, up to a high risk of bias (i.e., was rated very low). Meta-analyses were planned, although the reduced number of studies precluded its incorporation in the final manuscript. Only two from six studies that assessed balance found improvements after OCS compared to controls. No differences were found between OCS vs. control groups for fall risk or falls. The available evidence does not allow a definitive recommendation for or against OCS interventions as an effective strategy to improve balance and reduce the fall risk or falls in older adults. Therefore, more high-quality studies are required to draw definitive conclusions.

## 1. Introduction

Falls are the second leading cause of death from unintentional injuries worldwide [1], with the highest prevalence in adults ≥ 60 years of age [1,2]. Falls are related to several factors [2,3], with aging increasing the risk, which could be related to decreased muscle mass and strength, joint mobility, flexibility, balance, and agility [4], reduced physical function, and the ability to carry out activities of daily living independently. It has been observed that older adults with markers of frailty (e.g., under body mass index, cognitive impairment, disability) have up to 53% greater probability of recurrent falls [5]. On the other hand, balance, defined as a complex motor skill derived from the interaction of various sensorimotor processes in order to control the body in space [6], corresponds to a relevant factor since improvements in static, dynamic, reactive, or multitasking balance can reduce fall risk in healthy older adults [7].

The regular practice of physical activity (PA) favors the mobility and functional independence of older adults, in addition to improving physical functions (e.g., strength, gait stability, coordination, agility), mental health (e.g., self-esteem, quality of life) [8], and reduces the risk of cardiovascular and all-cause mortality. It has been reported that PA could also decrease the risk of developing some types of cancer, such as breast and prostate cancer, and generate healthier aging trajectories associated with metabolic benefits [9]. Systematic reviews have reported that older adults treated with PA of different types (i.e., resistance training, endurance training, multi-component training, balance training, exergames) show improvements in balance ranging from 16% to 24% [10] and a lower incidence of falls ranging between 22% and 58% [11,12] compared to control groups without PA.

In addition, the regular practice of PA has achieved greater diffusion in recent years as a strategy for fall prevention and improving or maintaining the general health of older adults living in the community [13,14] or participating in government programs [15], which contributes to an active and healthy lifestyle. The strategies to promote active and healthy aging need to be reassessed due to the COVID-19 pandemic to promote the safe practice of PA among older adults, considering, among other measures, practicing social distancing [16,17] or training at home [18]. Such a scenario could provide the opportunity to promote PA that requires a limited number of participants in reduced spaces for its practice, such as Olympic combat sports (OCS). In this context, OCS (boxing, fencing, judo, karate, taekwondo, and wrestling) are PA strategies that allow the development of sports practice individually or in pairs in confined spaces through low-impact dynamic actions with moderate to vigorous intensities using the upper and lower limbs through attack and defense movements, choreographies or specific forms of the disciplines and that can be practiced without contact [19,20]. Likewise, OCS include education about falls and exercises to face them [21] within their basic primary contents and achieve an adherence greater than 80% in older adults who practice OCS [19]. To the best of our knowledge, no review has attempted to summarize the currently available literature regarding the potential effects of OCS on balance, fall risk, or falls in older adults. Recent systematic reviews have reported improvements in physical–functional, physiological, and psychoemotional health in older adults who participated in interventions with OCS [19] and a better postural balance in adults who practiced hard martial arts such as taekwondo, judo, karate, soo bahkdo, and ving tsun, compared with groups that participated in dancing, football, running, walking, swimming, and cycling, or did not practice PA [20]. However, their results are not conclusive due to the diversity of data collection instruments reported by the studies [19] or due to half of the studies analyzed having a cross-sectional design [20], among other factors. In this aspect, a systematic review with a meta-analysis would allow the sample size of different studies to be aggregated and can provide not only high-quality evidence but also new insights for practitioners that can help them make better-informed, evidence-based decisions regarding the implementation of OCS [22]. Furthermore, a systematic review can help detect gaps and limitations in the scientific literature on OCS, providing valuable information for researchers. Therefore, the primary aim of this systematic review with meta-analysis was to assess the available body of published peer-reviewed articles related to the effects of OCS compared with active/passive controls on balance, fall risk, or falls outcomes in older adults.

## 2. Methods

### 2.1. Protocol and Registration

The conduct of this systematic review followed the preferred reporting guidelines for Systematic Review Protocols and meta-analyses PRISMA (Preferred Reporting Items for Systematic Reviews and Meta-analyses) [23]. The protocol was registered in PROSPERO (International Prospective Register of Systematic Reviews; code: CRD42021272133).

### 2.2. Eligibility Criteria

The inclusion criteria for this systematic review were original peer-reviewed articles without any restriction of language or publication date, published up to November 2021. Excluded records were conference abstracts, books and book chapters, editorials, letters to the editor, trial records, reviews, case studies, and essays. In addition, the framework of population, intervention, comparator, outcomes, and study design (PICOs) was followed to incorporate the studies into a systematic review (see Table 1).

### 2.3. Information and Database Search Process

The search process was performed between August and November 2021, using eight databases: PubMed, MEDLINE, Web of Science, Scopus, Cochrane Library, PsycINFO (American Psychological Association) for social and behavioral Sciences, CINAHL (Cumulative Index to Nursing and Allied Health Literature) complete and the collection of Psychology and Behavioral Sciences (EBSCO). The medical subject headings (MeSH) from the United States of America National Library of Medicine used bias-free language terms related to balance, fall risk, falls, OCS, and older adults. The search string used was the following: (“postural control” OR “postural balance” OR “balance” OR “postural equilibrium” OR “timed up and go” OR “timed up-and-go”) AND (“fall” OR “falls” OR “faller” OR “fell” OR “falling” OR “fall risk” OR “falls risk” OR “risk of falls” OR “fall rates” OR “accidental falls”) AND (“boxing” OR “fencing” OR “judo” OR “karate” OR “taekwondo” OR “wrestling” OR “Olympic combat sports”) AND (“elderly” OR “older adults” OR “older people” OR “older subject” OR “aging” OR “ageing” OR “aged”). The included articles and inclusion and exclusion criteria were sent to two independent experts to help identify additional relevant studies. We established two criteria that the experts had to meet: (i) to have a Ph.D. in sports sciences; and (ii) to have peer-reviewed publications on aging and/or OCS in impact factor journals according to Journal Citation Reports^®^. The experts were not provided with our search strategy to avoid skewing their searches. Once all of these steps were completed, on 2 November 2021, the databases were searched to retrieve relevant errata or retractions related to the included studies.

### 2.4. Studies Selection and Data Collection Process

The studies were exported to the EndNote references manager (version X8.2, Clarivate Analytics, Philadelphia, PA, USA). Two authors (PVB, THV) independently searched, eliminated duplicates, screened titles and abstracts, and analyzed full texts. No discrepancies were found at this stage. The process was repeated for searches within the lists of references and suggestions provided by external experts. Subsequently, potentially eligible studies were reviewed in full text, and reasons for excluding those that did not meet the selection criteria were reported.

### 2.5. Methodological Quality Assessment

The methodological quality of selected studies was assessed with the TESTEX [25], specifically designed for its use in physical exercise-based intervention studies. The TESTEX results were used as a possible exclusion criterion [25]. It has a 15-point scale (5 points for study quality and 10 points for reporting) [25]. This process was conducted independently by two authors (PVB, THV), and a third author (RRC) acted as a referee in the doubtful cases, which then were validated by another author (PVB).

### 2.6. Data Synthesis

The following data from the selected studies were obtained and analyzed: (i) author and publication year; (ii) country of origin; (iii) study design; (iv) initial health of the sample; (v) number of participants in the intervention and control groups; (vi) mean age of the sample; (vii) activities developed in the OCS groups and control groups; (viii) training volume (total duration, weekly frequency and time per session); (ix) training intensity; (x) data collection instruments of balance; (xi) data collection instruments of fall risk or falls; and (xii) the main outcomes of the studies.

### 2.7. Summary Measures for Meta-Analysis

Meta-analyses were included in the study protocol, with full details available at PROSPERO, registry code CRD42021272133. However, the low number of includable studies reporting data for the same outcome or similar testing procedures precluded a robust meta-analysis.

### 2.8. Certainty of Evidence

The studies were assessed for the certainty of evidence using the Grading of Recommendations, Assessment, Development and Evaluation (GRADE) scale [26] and were classified as having a high, moderate, low, or very low certainty of evidence. All analyses started with a grade of high certainty due to including studies with experimental design (randomized controlled trial and non-randomized controlled trial) and were downgraded if there were concerns over the risk of bias, consistency, precision, directness of the outcomes, or risk of publication bias [26]. Due to the small number of studies, the risk of publication bias was not assessed [27]. Two authors (PVB, THV) independently assessed the studies, and any discrepancies were resolved through consensus with a third author (RRC).

## 3. Results

### 3.1. Study Selection

**Figure 1:**
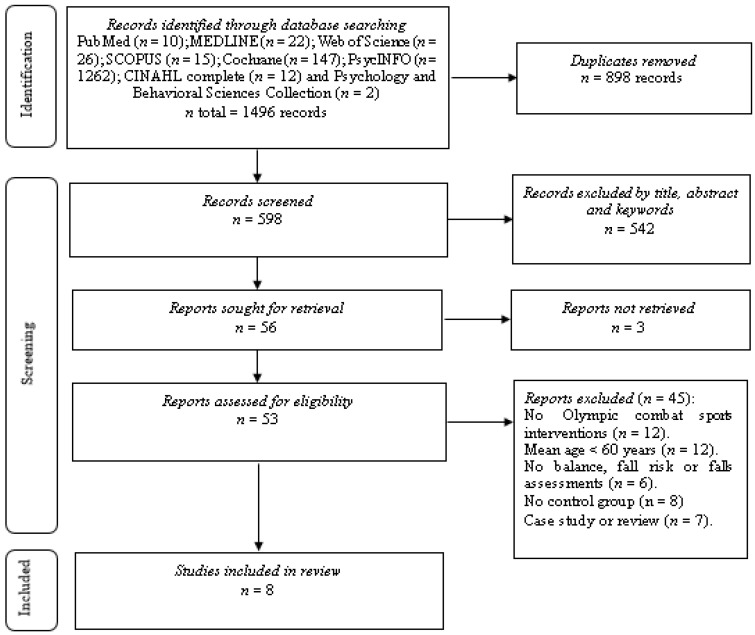
Flowchart of the review process. Legends: Based on PRISMA guidelines [23].

Figure 1 details the search process for the studies. A total of 1496 records were found in the study identification phase. Subsequently, duplicates were eliminated, and the studies were filtered by selecting the title, abstract, and keywords, resulting in 598 references. A total of 56 studies were included the next phase of the analysis, three of which were excluded as the texts were inaccessible (authors of inaccessible studies were contacted requesting a copy of their manuscript, estimating 30 days as a maximum response time). Fifty-three studies were analyzed via the full text, 12 of which were excluded due to not being OCS interventions, 12 due to the mean age of the sample being lower than 60 years, six for not having assessments of balance, fall risk, or falls, eight for not having a control group, and seven for being case studies or reviews. After this process, the total number of studies that met all selection criteria was eight [28,29,30,31,32,33,34,35].

### 3.2. Methodological Quality

The eight selected studies were analyzed using the TESTEX scale (Table 2). All of the studies achieved a score equal to or greater than 60% of the scale, specifically: 9/15 [29,33], 10/15 [28,35], 11/15 [30], 12/15 [31,34], and 13/15 [32], indicating a moderate to high methodological quality, so no studies were excluded from the systematic review.

### 3.3. Risk of Bias within Studies

The certainty of evidence was assessed with the GRADE scale. The risk of bias was considered with some concerns in four studies [30,32,34,35] and high risk in four studies [28,29,31,33]. Five studies presented some concerns for the randomization process [28,30,32,34,35] and three with high risks of bias not randomizing the groups [29,31,33]. Three studies were assessed with some concerns for missing outcomes due to not reporting the post-assessment for some outcomes [29,31,33], and five studies were rated as having a low risk of bias [28,30,32,34,35]. All studies were assessed with some concerns for selecting the reported result as there was no pre-registered protocol and a low risk of bias for deviations from intended intervention-effects of assignment to intervention and measurement of the outcome (Table 3).

### 3.4. Studies Characteristics

Table 4 summarizes the variables analyzed in each of the selected studies. Of these, three were developed in South Korea [28,30,35], two in the United States of America [32,33], one in Spain [29], one in Italy [31], and one in Germany [34]. Regarding the design of the studies, five were randomized controlled trials [28,30,32,34,35], and three were non-randomized controlled trials [29,31,33].

### 3.5. Sample Characteristics

One study had 24 participants [28], six had 30 to 40 [29,30,31,32,33,35], and one had 90 participants [34], totaling a sample of 322 older adults (64% female) with a mean age of 71.1 years. Regarding the initial health of the participants, seven studies report that the older adults included were people with no apparent health problems [28,29,30,31,33,34,35], which complements the groups of Campos-Mesa et al. [29], indicating that their participants are also pre-frail older adults. Only one study involved older adults with Parkinson’s disease [32]. Three studies indicated that their participants had no prior experience in OCS when initiating the interventions [30,31,33], and five studies did not report on their participants’ prior experience in OCS [28,29,32,34,35]. Five studies reported on the history or fall risk in older adults, and two of these indicated that 90% of the participants had suffered a fall in the past six months [33] or at some point before their fall inclusion in the study [29]. One study reported that the maximum number of falls in its participants was three last year [34]. Two studies indicated that their participants had a low fall risk according to the Berg Balance Scale (BBS) with mean scores of 49 [32] and 55 points [31]. Three studies did not report their participants’ history or fall risk [28,30,35].

### 3.6. Interventions Conducted and Dosing

Six studies had two groups of analysis [28,29,30,31,32,33]: an experimental group that participated in the intervention with OCS and a control group that maintained the usual activities of daily living [28,30,31,33] or participated in physical fitness programs which were focused on exercises and activities to develop endurance, muscle strength, cardiorespiratory fitness, flexibility, agility, and balance [29,32]. Two studies included three groups [34,35], one OCS group, one control group that maintained the usual activities of daily living, and a third group that, in the case of Witte et al. [34], participated in physical fitness (focused on activities to develop endurance, muscle strength, cardiorespiratory fitness, flexibility, agility, and balance) and in the case of Youm et al. [35], in walking exercise.

Concerning the activities developed in the OCS interventions, four studies used taekwondo [28,30,33,35], including technical foundations such as basic postures (short stride, long stride, and rider position), displacements (forward, backward, and side shifts), punches, blocks (low, medium, and high), and kicks (front, roundhouse, and descending), performed individually and in pairs, in addition to modality-specific choreographies or forms (Poomsae) [28,30,33,35]. Two studies applied judo training [29,31], including light routines and dynamic movements of the whole body, imitating judo techniques, followed by judo-specific passive and active standing and ground techniques, performed individually and in pairs, to end with choreography or specific forms of judo [29,31]. One study used boxing as an intervention modality [32], reporting specific cardiorespiratory fitness activities distributed as a training circuit, without detailing the exercises, but following recommendations from previous studies [36] and noting that the participants used gloves and punching bags without making contact with other people while boxing [32]. Meanwhile, Witte et al. [34] used karate as an intervention modality through various postures (forward, backward, and straddle legs), displacements with arm techniques during standing (fist blows, blocks, and finger blow), and kicking. In addition, the participants learned simple attack and defense exercises with their partners and a simple choreography or kata [34]. In our systematic review, no studies that used fencing or wrestling as an intervention modality for older adults were found.

Regarding the people who led the sessions with OCS, four studies reported that instructors or experienced professionals in the described modalities conducted them [29,30,31,32], while four studies did not state who was in charge of leading the sessions with OCS [28,33,34,35].

Regarding the duration of the interventions with OCS, one lasted for six weeks [29], six lasted between 11 and 16 weeks [28,30,31,32,33,35], and one lasted for 20 weeks [34]. Regarding the training frequency, seven studies reported a weekly distribution of two and three sessions [28,29,31,32,33,34,35], and one had five weekly sessions [30]. The duration of the sessions was 60 min [28,29,30,31,33,34,35], while one study reported a 90 min training session [32]. Three studies reported training intensity, two through the percentage of maximum heart rate (%HRmax), specifically between 40 and 60%HRmax [35] and between 50 and 80%HRmax [30], while Ciaccioni et al. [31], reported it as moderate to vigorous.

### 3.7. Data Collection Instruments of Balance

Four studies used the Timed Up-and-Go (TUG) test [28,30,32,33]. Furthermore, Combs et al. [32] used the Activities-specific Balance Confidence scale and Dual-task Timed Up-and-Go test, while Cromwell et al. [33] included the Gait Stability Ratio, which is where the length of time a participant stands on one leg is recorded with a stopwatch, alongside the Multidirectional Reach Test. For their part, Witte et al. [34] only considered the Motor Balance Test, and Youm et al. [35] measured variables of the center of pressure with a force platform by requesting the participants to keep their eyes open.

### 3.8. Data Collection Instruments of Fall Risk or Falls

Two studies used the Falls Efficacy Scale-International [29,31], which was complemented by Campos-Mesa et al. [29] with the World Health Organization (WHO) Questionnaire for the study of falls in the older adults. In addition, two studies used the BBS [31,32], while Cromwell et al. [33] used a dichotomous question about fear of falling.

### 3.9. Balance

Although the low number of includable studies reporting data for the same outcome precluded a robust meta-analysis, six studies reported balance as the main outcome, and four studies provided TUG data (i.e., time), involving four OCS and four control groups that were compared (pooled *n* = 132). The results showed similar changes in TUG after OCS compared to passive and active controls (ES = −0.27, small; 95% CI = -0.61 to 0.06; *p* = 0.112; I^2^ = 0.0%; Egger’s test, *p* = 0.281). On the other hand, when analyzing the individual results for TUG, two studies reported a significant decrease (*p* < 0.05) in the execution time in favor of the participants who intervened with OCS compared to control groups [28,33], and two studies showed no difference between the groups with OCS compared to control groups that maintained the usual activities of daily living [30] and participated in physical fitness [32].

In the case of Combs et al. [32], a significant increase (*p* = 0.015) in the total score of the Activities-specific Balance Confidence scale was reported in favor of the group with physical fitness compared to the group with OCS, but with no differences reported in Dual-task Timed Up-and-Go test in older adults with Parkinson’s disease. For their part, Cromwell et al. [33] reported a significant increase (*p* < 0.05) in the Multidirectional Reach Test and a significant decrease (*p* < 0.05) in the Gait Stability Ratio in favor of the group with OCS compared to the control (passive) group, without reporting differences in the time of standings on one leg. In the case of Witte et al. [34], there were no differences in the dynamic and static balance between the group with OCS vs. control group (passive), but there was a significant pre- and post-intervention improvement of the dynamic balance in the group with OCS (*p* = 0.015) and in the passive group (*p* = 0.018). The study by Youm et al. [35] reported a significant improvement in balance in the groups with OCS and walking exercise compared to the control group (passive) (*p* < 0.05), reflected by the decrease in variables such as the root mean square, speed, and area of the center of pressure both in the mediolateral and anteroposterior directions. In summary, of the six studies analyzed, two reported favorable balance changes for the groups with OCS vs. passive groups [28,33].

### 3.10. Fall Risk or Falls

Four studies reported a fall risk or falls as the main outcome. Although two studies used the BBS to assess older adults [31,32], only one reported post-intervention results. No differences were found between the group with OCS vs. the group with physical fitness for the BBS, but there were significant improvements (*p* = 0.005) pre- and post-intervention independently in both groups of older adults with Parkinson’s disease [32]. Two studies were evaluated with the Falls Efficacy Scale-International; on the one hand, Campos-Mesa et al. [29] reported a significant decrease (*p* = 0.002) in fear of falling in favor of the group intervened with OCS compared to the group with physical fitness, while Ciaccioni et al. [31], did not achieve significant changes in the Falls Efficacy Scale-International. Finally, two assessments were not used to measure post-intervention, specifically, the WHO Questionnaire [29] and the dichotomous question on fear of falling [33]. In summary, no differences were found between the groups that intervened with OCS vs. the control groups of the four studies that reported fall risk or falls.

### 3.11. Certainty of Evidence

The limitations related to the certainty of evidence prevent a recommendation for or against the use of OCS as interventions that favor balance, reduce fall risk, or reduce falls in older adults (Table 5).

### 3.12. Adverse Events and Adherence

The analyzed studies did not report adverse events (injuries) in older adults who participated in the OCS interventions. Three studies did not report attrition in judo [29] and taekwondo [33,35] interventions regarding adherence. Four studies achieved adherence equal to or greater than 84% in interventions with taekwondo [28,30], judo [31], and karate [34]. Only one study reported a 64% adherence to boxing [32]. The main reasons for attrition were personal problems, not complying with the minimum attendance at training sessions, or an unsupported schedule [28,29,31,32,33,35]. One study added the loss of interest of older adults with Parkinson’s disease to these reasons [32], while two studies did not report the reasons for attritions [30,34].

## 4. Discussion

The present systematic review aimed to evaluate the effectiveness of OCS on balance, fall risk, or falls in older adults. The systematic review identified eight studies with a methodological quality ≥ 60% of the established score. However, the certainty of evidence was rated very low. Therefore, it is impossible to establish a definitive recommendation for or against OCS interventions as an effective strategy to improve balance, reduce fall risk, or falls in older adults.

Regarding balance, it was only possible to perform a meta-analysis of the TUG data [28,30,32,33] without finding changes in favor for or against the groups with OCS vs. active or passive control groups (ES = −0.27). Individual studies reported significant improvements (*p* < 0.05) in the groups that intervened with OCS at the time of the TUG test [28,32,33], Gait Stability Ratio [33], variables of the center of pressure [35], Activities-specific Balance Confidence scale [32], and dynamic balance [34]. It has been suggested that one of the factors that would cause a deterioration in balance in older adults would be lower functionality of the lower limbs due to a loss of muscle mass and strength, the aging of biomechanical elements of the musculoskeletal system, a decreased range of motion of the ankle, and less elasticity of the foot tissues, which would limit sensory feedback [13]. Furthermore, poor balance in older adults has been associated with a delay in muscular responses triggered by proprioceptive deficits that are part of the aging process [37]. Therefore, practicing PA that involves sensorimotor stimuli could favor balance [10,20] through different training methods, such as soft [38] and hard [20] martial arts.

Four of the analyzed studies evaluated the fall risk, or falls, using the BBS [31,32], Falls Efficacy Scale-International [29,31], WHO Questionnaire [29] or a dichotomous question about fear of falling [33]; in some cases, it was assessed post-intervention [29,33]. However, two studies reported a significant reduction (*p* < 0.01) in the fall risk [32] and a fear of falling [29]. The cause for falls are multifactorial [2,3], requiring a multidimensional intervention for fall prevention [2]. In addition, it has been indicated that balance, mobility, medication, and psychological, sensory, and neuromuscular aspects are predictive factors of recurrent falls in older adults [5]. In this sense, reducing the fall risk and fear of falling through OCS is auspicious since it has been proposed as a simple and inexpensive solution to teach people about falling safely as early as possible in their lives [21] and in an elementary content in most OCS.

On the other hand, the results of our systematic review are aligned with previous systematic reviews that have reported some improvements and the maintenance of balance in adults who practice hard martial arts [20] and significant improvements (*p* = 0.017) in lower limb strength measured through the chair stand test in older adults in the intervention with OCS group compared to control groups without PA [19]. Muscle strength is a physical quality that has been linked to a more outstanding balance and lower fall risk in aging [4,11], which is considered a basic element in programs focused on fall prevention in older adults [10,14]. Therefore, it is essential to promote PA among older adults, which focuses on the strengthening of the large muscle groups, cardiorespiratory fitness, and balance [15], since an adverse effect has been seen in balance that ranges from 14.9% to 21.9% in groups without PA [10] and a decrease in falls (31–35%) in older adults participating in community PA programs compared to passive control groups [14]. In this sense, OCS may improve older adults’ well-being and general health status [19], muscle strength, cardiorespiratory fitness, and balance. In addition, OCS interventions involve education about falls, including exercises to prevent and avoid adverse effects during falls (i.e., fall-technique drills) [21]. For these reasons, the OCS would positively impact older adults’ functional independence and quality of life [19,39].

Regarding the certainty of evidence, our systematic review reported it as very low, without allowing us to establish definitive recommendations, a situation that coincides with a meta-analysis focused on resistance training as a method for fall prevention in older adults [12]. Similarly, systematic reviews with soft martial arts such as tai chi in older adults [38] and with hard martial arts in adults [20] have reported that the methodological quality of the studies selected is moderate to low. Therefore, they recommend considering their results with caution. In contrast, bibliometric reviews with OCS have reported a significant number of studies published in the Web of Science; for example, judo was reported in 383 indexed articles between 1956 and 2011 [40], and taekwondo in 340 articles between 1988 and 2016 [41]; despite the increasing productivity on OCS, the certainty of evidence of the studies found in our systematic review is low. Future studies with OCS should use double-blind randomization and supervised control groups among other methodological strategies, as well as reporting all post-intervention results, and previously register their research protocols [26], which would help to improve the quality of their research designs and, consequently, the certainty of evidence.

Regarding the dosage used for the OCS interventions, average values of 12 weeks were presented, with two to three weekly sessions of 60 min, while three studies reported the intensity as using 40–80%HRmax [30,35] or moderate to vigorous activity [31]. The frequency and intensity reported by the studies with OCS align with the international PA recommendations for older adults [4,42]. The activities selected in the OCS interventions were based on basic technical foundations of the disciplines (boxing, judo, karate, and taekwondo); no adverse effects were reported, and they achieved a mean adherence >80%. With the appropriate dosage and selection of activities, OCS can meet the PA level that older adults need each day [19]. Otherwise, four of the selected studies reported that the sessions with OCS were led by instructors or experienced professionals in the described modalities [29,30,31,32]. Having instructors or experienced professionals in OCS can guarantee safe practice due to the high incidence of injuries that their sports practice (elite competition) generates, being the most recurrent in the head (45.8%) for boxing, in the knees (24.8%) for wrestling, in the fingers (22.8%) for taekwondo and in the lower back (10.9%) for judo [43]. Also, due to the diversity of technical foundations (e.g., postures, displacements, blows, projections, kicks) that make up the contents of the OCS, this negates the higher risk of injury due to poor execution and requires adaptations to be made to the activities so that older adults can perform them; therefore, permanent training of instructors or professionals in charge of directing training with combat sports has been suggested to achieve the greatest benefits [44]. In this sense, previous systematic reviews have suggested selecting combat sports according to people’s interests and initial health statuses [19,20], as well as non-contact activities, practiced individually or in pairs through choreographies or forms according to the level of experience in OCS and the age range of older adults [19], respecting the basic training principles such as progressive overload with a moderate to vigorous intensity and a frequency of two to three weekly sessions of 60 min [19], for safe practice. The background is aligned with what was reported in the studies analyzed in our systematic review. The main limitation of our systematic review is the low certainty of evidence found, similar to previous systematic reviews in the field of martial arts and combat sports [20,38]. In addition, the low number of includable studies reporting data for the same outcome or with similar testing procedures precluded a robust meta-analysis for the effects of OCS on balance, fall risk, or falls. The facts show that OCS practice in older adults is an emerging field that needs more support and research [19]. Considering the responses obtained in the present systematic review, OCS practice in older adults could be safe and relevant to improving health, given the studies discussed. In addition, OCS are low-cost and affordable PA strategies, requiring reduced space, little implementation, and motivating older adults due to the diversity of technical foundations and activities carried out [19,20,21]. However, due to the heterogeneity of the studies included in this systematic review, it is impossible to establish a definitive recommendation for OCS interventions to improve balance and reduce the fall risk or number of falls in older adults. As acknowledged by the authors, no studies compared individuals who started OCS after 60 years of age with individuals who practiced OCS during several stages of life. The responses of these studies may be different among participants who started OCS in the aging phase compared to the older adults who had practiced OCS since adolescence and young adulthood. Such responses may be different due to the stimulus performed, i.e., frequency, volume, intensity, density, and other physical exercises conducted in parallel (e.g., weightlifting, endurance training, high-intensity interval training), which would probably help develop physical fitness. The sample size of the studies with OCS often uses competitive athletes or physically active people seeking health and quality of life or recreation. Due to this, the comparison and direction of our systematic review findings remain limited with which to provide an accurate diagnosis, promoting only speculations.

## 5. Conclusions

The available evidence does not allow a definitive recommendation for or against OCS interventions as an effective strategy to improve balance and reduce the fall risk or falls in older adults. Therefore, more high-quality studies are required to draw definitive conclusions.

## Figures and Tables

**Table 1:** Selection criteria used in the systematic review.

Category	Inclusion Criteria	Exclusion Criteria
Population	Older adults, considered as older adult participants with an average age of 60 years or more according to the World Health Organization [24], and without distinction of sex.	People under 60 years of age.
Intervention	Interventions with Olympic combat sports (boxing, fencing, judo, karate, taekwondo, wrestling) lasting four weeks or more.	Exercise interventions not involving Olympic combat sports.
Comparator	Interventions that had a control group with or without supervised physical activity.	Absence of control group.
Outcome	At least one balance assessment (e.g., force platform, timed up-and-go test), fall risk, or falls assessment (e.g., Falls Efficacy Scale-International, Berg Balance Scale) before and after the intervention.	Lack of baseline and/or follow-up data.
Study design	Studies with experimental design (randomized controlled trial and non-randomized controlled trial) with pre- and post-assessment.	Cross-sectional, retrospective, and prospective studies.

**Table 2:** Study quality assessment according to TESTEX scale.

Study	Eligibility Criteria Specified	Randomly Allocated Participants	Allocation Concealed	Groups Similar at Baseline	Assessors Blinded	Outcome Measures Assessed >85% of Participants *	Intention to Treat Analysis	Reporting of between Group Statistical Comparisons	Point Measures and Measures of Variability Reported **	Activity Monitoring in the Control Group	Relative Exercise Intensity Reviewed	Exercise Volume and Energy Expended	Overall TESTEX #
Baek et al. [28]	Yes	Yes	Unclear	Yes	Unclear	Yes (2)	Yes	Yes	Yes (2)	No	No	Yes	10/15
Campos-Mesa et al. [29]	Yes	No	Unclear	Yes	Unclear	Yes (1)	Yes	Yes	Yes (2)	Yes	No	Yes	9/15
Cho & Roh [30]	Yes	Yes	Yes	Yes	Unclear	Yes (2)	Yes	Yes	Yes (1)	No	Yes	Yes	11/15
Ciaccioni et al. [31]	Yes	No	Yes	Yes	Unclear	Yes (3)	Yes	Yes	Yes (2)	No	Yes	Yes	12/15
Combs et al. [32]	Yes	Yes	Yes	Yes	No	Yes (3)	Yes	Yes	Yes (2)	Yes	No	Yes	13/15
Cromwell et al. [33]	Yes	No	Unclear	Yes	Yes	Yes (2)	Yes	Yes	Yes (1)	No	No	Yes	9/15
Witte et al. [34]	Yes	Yes	Yes	Yes	No	Yes (3)	Yes	Yes	Yes (2)	No	No	Yes	12/15
Youm et al. [35]	Yes	Yes	Unclear	Yes	Unclear	Yes (1)	Yes	Yes	Yes (2)	No	Yes	Yes	10/15

* Three points are possible: one point if adherence >85%, one point if adverse events are reported, one point if exercise attendance is reported. ** Two points are possible: one point if the primary outcome is reported, and one point if all other outcomes are reported. # total out of 15 points. TESTEX: Tool for the assEssment of Study qualiTy and reporting in EXercise [25].

**Table 3:** Risk of bias within studies.

Study	1	2	3	4	5	Overall GRADE
Baek et al. [28]	Some concerns	Low risk	Low risk	Low risk	Some concerns	High risk
Campos-Mesa et al. [29]	High risk	Low risk	Some concerns	Low risk	Some concerns	High risk
Cho & Roh [30]	Some concerns	Low risk	Low risk	Low risk	Some concerns	Some concerns
Ciaccioni et al. [31]	High risk	Low risk	Some concerns	Low risk	Some concerns	High risk
Combs et al. [32]	Some concerns	Low risk	Low risk	Low risk	Some concerns	Some concerns
Cromwell et al. [33]	High risk	Low risk	Some concerns	Low risk	Some concerns	High risk
Witte et al. [34]	Some concerns	Low risk	Low risk	Low risk	Some concerns	Some concerns
Youm et al. [35]	Some concerns	Low risk	Low risk	Low risk	Some concerns	Some concerns

1: randomization process. 2: deviations from intended intervention-effects of assignment to intervention. 3: missing outcome. 4: measurement of the outcome. 5: selection of the reported result. GRADE: Grading of Recommendations, Assessment, Development, and Evaluation. Low risk: low risk of bias. High risk: high risk of bias.

**Table 4:** Studies reporting on the effectiveness of Olympic combat sports on balance, fall risk, or falls in older adults.

Study	Country	Study Design	Sample’s Initial Health	Groups	Mean Age (Year)	Activities in the Intervention and Control Groups	Training Volume			Training Intensity	DCI of Balance	DCI of Fall Risk or Falls	Main Outcomes
				(n)			TD (Weeks)	Fr	TPS (min)				
								(Weekly)					
Baek et al. [28]	South Korea	RCT	Apparently healthy								TUG	None	EG vs. CG: ↓ TUG (in favour EG).
				EG: 12	72.6	EG: taekwondo	12	3	60	NA			
						
				CG: 12	72.4	CG: usual activities		NA	NA	NA			
						
Campos-Mesa et al. [29]	Spain	NRCT	Apparently healthy								None	FES-I	EG vs. CG: ↓ FES-I ^π^ (in favour EG).
								
				EG: 19	74.3	EG: judo	6	2	60	NA		WHO Questionnaire	WHO Questionnaire was not reported post-intervention.
								
				CG: 11	77.8	CG: physical fitness		2	60	NA			
Cho & Roh [30]	South Korea	RCT	Apparently healthy							50–80%HRmax	TUG	None	
				EG: 19	68.9	EG: taekwondo	16	5	60				
													EG vs. CG: ↔ TUG.
				CG: 18	69	CG: usual activities		NA	NA				
						
Ciaccioni et al. [31]	Italy	NRCT	Apparently healthy							Moderate to vigorous	None	FES-I	EG vs. CG: ↔ FES-I ^π^.
				EG: 19	69.3	EG: judo	16	2	60				
												BBS	BBS was not reported post-intervention.
				CG: 21	70.2	CG: usual activities		NA	NA				
							
Combs et al. [32]	United States of America	RCT	Parkinson’ ‘s disease									BBS	
				EG: 17	66.5	EG: boxing	12	2–3	90	NA	ABC TUG		EG vs. CG: ↑ ABC (in favour CG), ↔ BBS, ↔ TUG, ↔ DTUG.
											DTUG		
				CG: 14	68	CG: physical fitness		2–3	90	NA			
								
Cromwell et al. [33]	United States of America	NRCT	Apparently healthy									One question	
				EG: 20	72.7	EG: taekwondo	11	2	60	NA	TUG		EG vs. CG: ↓ TUG, ↑ MDRT, ↓ GSR, ↔ SLS (in favour EG).
											MDRT		
				CG: 20	73.8	CG: usual activities		NA	NA	NA	GSR		One question was not reported post-intervention.
											SLS		
								
Witte et al. [34]	Germany	RCT	Apparently healthy								MBT (static and dynamic balance)	None	EG vs. FG vs. CG: ↔ Static balance, ↔ Dynamic balance.
				EG: 30		EG: karate		2	60	NA			
						
				FG: 30	69.3	FG: physical fitness	20	2	60	NA			
						
				CG: 30		CG: usual activities		NA	NA	NA			
						
						
Youm et al. [35]	South Korea	RCT	Apparently healthy				12					None	EG vs. WG vs. CG: ↔ AP RMS distance, ↔ AP velocity, ↔ AP total power frequency, ↓ ML RMS distance, ↓ ML velocity, ↔ ML total power frequency (in favour EG and WG regarding CG).
				EG: 10	69.4	EG: taekwondo		3	60				
				WG: 10	71.4	WG: walking exercise		3	60	40–60%HRmax	Force Platform (COP)		
				CG: 10	70.6	CG: usual activities		NA	NA				
						
						
						

ABC: Activities-specific Balance Confidence Scale. AP: anteroposterior. BBS: Berg Balance Scale. CG: control group. COP: center of pressure. DCI: data collection instruments. DTUG: Dual-task timed up-and-go test. EG: experimental group. FES-I: Falls Efficacy Scale-International. FG: fitness group. Fr: frequency. GSR: Gait stability ratio. n: number of participants. MBT: motor balance test. MDRT: Multidirectional Reach Test. ML: mediolateral. NA: not applicable. NRCT: a non-randomized controlled trial. RCT: a randomized controlled trial. RMS: root mean square. SLS: the length of time a participant stood on one leg. TD: total duration. TPS: time per session. TUG: timed up-and-go test. %HRmax: percentage of maximum heart rate. ↔: no significant difference. ↑: significant increase. ↓: significant decrease. ^π^: overall score.

**Table 5:** GRADE assessment for the certainty of evidence.

Outcomes	Study Design	Risk of Bias in Individuals Studies	Risk of Publication Bias	Inconsistency	Indirectness	Imprecision	Certainty of Evidence	Recommendation
Balance	5 RCTs and 1 NRCTs with a 9 trials and 252 participants	Moderate to High ^1^	Not assessed ^3^	Moderate ^4^	Low ^5^	High ^6^	Very low ^7^	The OCS does not show superior effects in the older adults compared to control groups (active/passive) on balance, fall risk, or falls.
Fall risk or falls	1RCT and 2 NRCTs with a 5 trials and 91 participants	Moderate to High ^2^	Not assessed ^3^	Moderate ^4^	Low ^5^	High ^6^	Very low ^7^	

GRADE: Grading of Recommendations, Assessment, Development, and Evaluation. NRCT: a non-randomized controlled trial. OCS: Olympic combat sports. RCT: a randomized controlled trial. ^1^ Four studies with some concerns and two with high overall risk of bias. ^2^ One study with some concerns and two with high overall risk of bias. ^3^ Not assessed due to the small number of studies. ^4^ High statistical heterogeneity (as assessed through I2) and/or high clinical or methodological heterogeneity (interventions and study designs, respectively). ^5^ Balance and fall risk or falls had to be directly measured in our study, thereby not using surrogate outcomes. The population (older adults) was clearly defined and corresponded to our goals. ^6^ Very large 95% confidence intervals. ^7^ Moderate to high risk of bias, lack of risk of publication bias assessment, moderate inconsistency, low indirectness, and high imprecision resulted in very low certainty of evidence.

## Data Availability

The datasets generated during and/or analysed during the current review are available from the Corresponding author upon reasonable request.
